# Preliminary Report on the Time-Related Effect of a Single Autologous Adipose-Derived Mesenchymal Stem Cells Injection in Hip Osteoarthritis: A Retrospective Observational Study

**DOI:** 10.1155/aort/3424035

**Published:** 2025-06-16

**Authors:** Adriano Braile, Annalisa De Cicco, Sara Liguori, Vincenzo De Matteo, Gianluca Conza, Michele Vasso, Maria Consiglia Trotta, Giuseppe Toro, Umberto Tarantino

**Affiliations:** ^1^Department of Medical and Surgical Specialties and Dentistry, University of Campania “Luigi Vanvitelli”, Naples, Italy; ^2^Department of Clinical Sciences and Translational Medicine, University of Rome Tor Vergata, Rome, Italy; ^3^Department of Orthopedics and Traumatology, Federico II Hospital, Naples, Italy; ^4^Knee Surgery and Sports Traumatology Unit, “Mater Dei” Private Clinic, Rome, Italy; ^5^Department of Experimental Medicine, University of Campania “Luigi Vanvitelli”, Naples, Italy

**Keywords:** adipose-derived mesenchymal stem cells, early osteoarthritis, hip osteoarthritis, intra-articular injection, regenerative medicine, stem cells

## Abstract

**Background:** Recently, intra-articular injection of mesenchymal stem cells (MSCs) had been proposed as a conservative treatment for hip osteoarthritis (HOA). Adipose tissue was demonstrated as a viable source of MSCs because of the high concentration of cells and the easy access to the donor site. The purpose of this study was to evaluate the time-related results of a single intra-articular injection of autologous adipose-derived stem cells (aASCs) in a series of patients with HOA.

**Methods:** A retrospective study was conducted on 30 patients with HOA, who underwent an intra-articular injection of aASCs between September 2018 and January 2021. Inclusion criteria for the procedure were as follows: onset of symptoms of the affected hip in the prior six or more months ago, failure of the conservative treatment (NSAIDs and/or physiotherapy) and age > 18 years. Exclusion criteria were trauma in the affected hip occurred in the previous 3 months, recent arthroscopic treatment, infectious joint disease, chondromatosis of the hip or any other secondary HOA, malignancy, hyaluronic acid or other injections in the previous 6 months and incomplete follow-up. Because a low BMI makes extremely difficult to harvest enough adipose tissue, patients with a BMI < 18 were also excluded. The Oxford Hip Score, the 12-item Short Form Survey and Visual Analogue Scale were used to evaluate the results of the proposed treatment at regular intervals.

**Results:** In 27/30 patients, a constant improvement in pain relief, hip function and quality of life was observed during the entire follow-up period of 12 months. Two patients underwent a subsequent total hip arthroplasty.

**Conclusion:** The single injection of aAMSCs seems to be a valuable treatment for HOA. A constant amelioration of pain and function could be observed in most patients at 12 months of follow-up.

## 1. Introduction

Hip osteoarthritis (HOA) is the second most common form of osteoarthritis (OA) after knee OA and affects 400 million people worldwide, with the highest prevalence in Europe (12.59%), followed by North America (7.95%), Asia (4.26%) and Africa (1.20%) [1, 2]. HOA is characterised by articular cartilage destruction and reactive bone changes, causing pain, reduction of joint function and patient mobility, thus impairing daily quality of life [3, 4]. HOA present a high impact on public healthcare services, considering the high costs (estimated to be between 500$–800$/yearly per patient) and the high prevalence [5]. The greatest impact is observed properly in later stages of HOA (i.e.,: Kellgren–Lawrence 4th stage) during the last year before total hip arthroplasty (THA) implantation [5]. This is a surgical procedure with good outcomes regardless of the age of implantation [6, 7]. However, THA is not without complications, including instability, infection, fractures and aseptic loosening [8–14]. Therefore, early HOA conservative management is often preferred [15]. Several methods have been proposed for the symptomatic treatment of early HOA, including rehabilitation protocols, nonsteroidal anti-inflammatory drugs (NSAIDs) and intra-articular injections [15]. Although effective, corticosteroid injections have shown to be effective in symptomatic HOA by decreasing pain only for few weeks [16]. Recently, regenerative medicine principles have been applied for the treatment of early OA, with the purpose of both slowing down the course of the disease and improving patients' function and pain. Among the regenerative therapeutic strategies explored for OA management, a viable option aimed to favour OA cartilage repair is represented by tissue engineering and regenerative approaches based on bioactive molecules, biomaterials and cells. To this regard, some methods such as chondrocyte transplantation, autologous chondrocyte implantation (ACI), matrix-associated autologous chondrocyte implantation (MACI) and microfractures (MF) showed several limitations due to variable success, clinical effectiveness, inconsistent cartilage repair and rate of complications [17]. Moreover, to date, data on the safety and efficacy of these techniques are further limited by the lack of long-term follow-up studies [17]. Less invasive biological approaches for the treatment of HOA are represented by the intra-articular injections of hyaluronic acid (HA), platelet-rich plasma (PRP) and stem cells [18]. Particularly, HA seems to be found effective in OA both reducing pain and improving joint lubrication [19]. Some of these effects might be related to the biological activities of the injected HA, represented by the increase in chondrocyte-mediated endogenous HA and proteoglycan synthesis; prevention of further cartilage degradation while promoting its regeneration; reduction in joint concentration of matrix metalloproteinases and proinflammatory mediators and reducing the pain sensitivity [20]. Similarly, PRP infiltrations seem to represent a useful treatment to reduce pain and improve function and the quality of life in OA patients, by providing the delivery of autologous growth factors which aid cartilage repair, thus representing a secondary level conservative procedure in knee OA [21–24]. However, considering the conflicting results associated with both procedures, a clear recommendation on their use in HOA is not available yet [25]. Recently, considering their regenerative properties, stem cells have emerged as the most intriguing therapeutic option in orthopaedics. In particular, their use would be potentially able to address the unmet medical needs for the degenerative processes underlying OA. Of note, while the use of induced pluripotent stem cells (iPSCs) raised major concerns regarding the safety in clinics, considering their immunogenicity and genomic instability, adult stem cells (i.e., haematopoietic and mesenchymal populations) showed great safety and efficacy in several diseases, with encouraging outcomes in clinical practice [26]. Although some attempts to assess the effect of haematopoietic stem cells (HSCs) in OA patients, the intrinsic ability of mesenchymal stem cells (MSCs) to differentiate into chondrocytes has increased the interest around them for the treatment of OA, also considering their wide spectrum of paracrine and endocrine effects [27, 28]. Adipose-derived mesenchymal stem cells (AMSCs) are suitable to treat HOA thanks to some potential advantages over alternatives such as PRP and HA. Autologous adipose-derived mesenchymal stem cells (aAMSCs) are known for their regenerative and anti-inflammatory capabilities, making them particularly effective at counteracting the cartilage degeneration and inflammation associated with OA. Unlike PRP, which mainly targets inflammation and can be beneficial for pain relief, aAMSCs have the potential to promote cartilage repair, which is crucial in halting OA progression. This regenerative potential arises from their ability to differentiate into chondrocytes and release paracrine factors that support tissue repair and modulate immune responses within the joint [29]. Moreover, adipose tissue has been considered one of the most promising tissues because of the high concentration of MSCs, the easy harvesting and good safety profile of the donor site [30–32]. Moreover, AMSCs showed a good safety and efficacy profile when applied in different musculoskeletal disorders, including several clinical settings of knee OA [33]. However, only few preliminary results are available regarding their efficacy and safety in patients with HOA. Therefore, the purpose of this study was to report the time-dependent effects of a single intra-articular injection of aAMSCs in a series of patients with HOA. Our hypothesis was that the use of aAMSCs in HOAs significantly improves clinics and patients' quality of life over the time of follow-up.

## 2. Materials and Methods

We conducted a STROBE-compliant retrospective study [34] on patients with HOA and who underwent a single injection of aAMSCs, between September 2018 and January 2021 at the University of Campania “Luigi Vanvitelli”. The diagnosis of HOA was made following the American College of Rheumatology (ACR) criteria and classified according to Kellgren–Lawrence system [35–37]. According to the ACR criteria, a patient was classified as having HOA if pain was present in combination with either (1) hip internal rotation ≥ 15°, pain present on internal rotation of the hip, morning stiffness of the hip for ≤ 60 min and age > 50 years, or (2) hip internal rotation < 15° and an erythrocyte sedimentation rate (ESR) ≤ 45 mm/hour; if no ESR was obtained, hip flexion ≤ 115° was substituted [37]. Inclusion criteria for the procedure were as follows: onset of symptoms of the affected hip in the prior six or more months ago, failure of the conservative treatment (NSAIDs and/or physiotherapy) and age > 18 years. Exclusion criteria were trauma in the affected hip occurred in the previous 3 months, recent arthroscopic treatment, infectious joint disease, chondromatosis of the hip or any other secondary HOA, malignancy, HA or other injections in the previous 6 months and incomplete follow-up. Because a low BMI makes extremely difficult to harvest enough adipose tissue, patients with a BMI < 18 were also excluded. All patients underwent clinical and standard X-ray evaluations before the procedure. Our indications for the procedure are summarised in Table 1. The aAMSCs were concentrated from the abdominal fat tissue as previously reported [38, 39], using the Lipogems Ortho kit (Lipogems International SpA, Milan, Italy). At least 5 mL of processed tissue was injected into the affected hip under fluoroscopic guidance, using the standard anterolateral portal for hip arthroscopy. Weight-bearing and complete hip range of motion (ROM) were allowed since the first day after the procedure. An abdominal compression binder was applied in the following 15 days to prevent effusion of the donor site. Moreover, for the first 7 days after the procedure, etoricoxib 60 mg is administered daily to reduce discomfort in both donor and injection sites. After that period, patients are allowed to take their usual therapy (i.e., paracetamol) in case of intractable pain. All patients were evaluated at regular intervals (3, 6 and 12 months). Primary endpoints were the improvement in pain and function, assessed using the Visual Analogue Scale (VAS) and the Oxford Hip Score (OHS), respectively. Secondary endpoints were improvement in quality of life (using the 12-item Short Form Survey [SF-12]) and rate of failure/complications. Any need for further joint injection/subsequent THA were considered as a failure of the procedure. Descriptive statistics were used to describe patients' demographics. To better report time-related effects, a one-way repeated measure analysis of variance (ANOVA), followed by Tukey's multiple comparison test, was performed using GraphPad Prism 6.0 software (La Jolla, CA, United States). The statistical significance was set at *p* < 0.05. Our institutional board approved the collection of the data reported in the present study in the study protocol n. 0035781/i. All patients provided written informed consent allowing them to undergo surgery and to have their data collected for scientific and audit purposes. The present study has been performed in accordance with the ethical standards as laid down in the 1964 Declaration of Helsinki and its later amendments or comparable ethical standards.

## 3. Results

**Figure 1:**
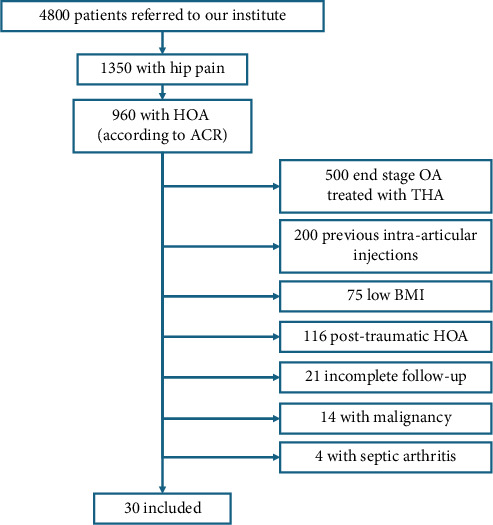
Patients' selection process during the inclusion period.

**Figure 2:**
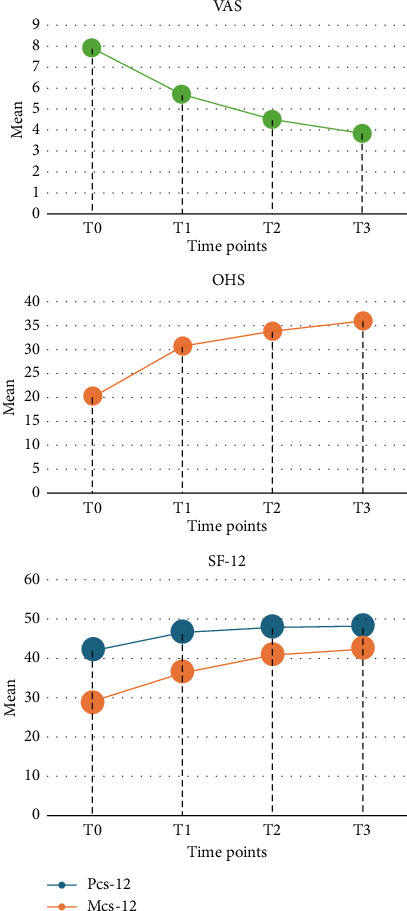
Mean Visual Analogue Scale (VAS), Oxford Hip Score (OHS) and SF-12 score from a preoperative value (T0) to postoperative 12-month follow-up (T3).

During the inclusion period, a series of 30 nonconsecutive patients were available to be analysed in the present study. Patients' selection process is summarised in Figure 1. The preoperative demographic data are reported in Table 2. In 27/30 patients the mean OHS, VAS and SF-12 scores improved constantly throughout the entire follow-up period (see Tables 3 and 4, and Figure 2) and were significantly increased if compared to preoperative values (see Table 3). Particularly, the OHS improved from 20.07 (range 15–29) preoperatively to 35.92 (range 20–55) at 12 months of follow-up (*p* value < 0.0001 vs. preoperative and *p* value < 0.05 vs. 3-month of follow-up), the VAS improved from 7.90 (range 6–9) to 3.8 (range 2–5) (*p* value < 0.05 vs. preoperative) and the mean SF-12 mental score improved from 42.28 (range 22.56–60.53) to 48.01 (range 34,97 to 61.90) (*p* value < 0.05 vs. preoperative), while the mean SF-12 physical score improved from 28.97 (range 15.19–46.06) to 42.43 (range 34.51–56.02) (*p* value < 0.0001 vs. preoperative and *p* value < 0.01 vs. 3-month of follow up). In three patients (10%), the single injection of aAMSCs failed after a mean of 8.6 months. In two patients, a subsequent THA was needed, whereas in the third patient, a new injection was performed (see Table 4 for further details). Regarding the satisfaction evaluation, 15 patients reported a Grade 5, nine a Grade 4, three a Grade 3, one a Grade 2 and two a Grade 1. A discomfort at the site of harvesting was reported by two patients and a transient cruralgia by another one. No serious complications were reported.

## 4. Discussion

The primary finding of this retrospective study was that a single injection of aAMSCs in selected patients with HOA was associated with good clinical outcomes and high patients' satisfaction, with a constant amelioration of pain, function and quality of life throughout the entire follow-up period. Theoretically, these results could be related to the paracrine and endocrine effects of aAMSCs [14, 40]. In fact, these cells secrete a huge variety of substances with several functions, including proangiogenic, antifibrotic, antiapoptotic and immunomodulatory effects in target tissues, such as the joint. [41]. The characterisation of the aAMSC secretome evidenced a high content of transforming growth factor beta 1 (TGFβ1), a potent inducer of cartilage extracellular matrix (ECM) synthesis, enhancing the production of cartilage-specific molecules such as collagen Type II and aggrecan [42, 43]. The aAMSC secretome could be crucial in OA joints, characterised by a proinflammatory milieu with a high rate of catabolic processes [29]. The anabolic activities favoured by aAMSCs potentially prevent further degeneration of the cartilage [44]. Furthermore, aAMSC secretome contains several exosomes (aAMSC-exos) with demonstrated cartilage-sparing molecules [45]. These growth factors are crucial for cartilage regeneration but are not exclusively derived from adipose tissue. Similar substances could be isolated from other biological sources, including PRP and bone marrow-derived mesenchymal stem cells (BM-MSCs) [46–48]. BM-MSCs are also widely used in orthopaedics and traumatology [49–55]. However, Tremolada et al., comparing lipoaspirate with concentrated bone marrow, demonstrated that the adipose tissue contains a greater density of MSCs than bone marrow [56]. Recently, several retrospective observational studies supported the use of aAMSCs in early OA, reporting promising results regardless of the joint affected [38, 41, 57]. Particularly, Panni et al., in a retrospective study on 52 patients with early knee OA treated with arthroscopic debridement plus intra-articular injections of aAMSCs, reported a significative improvement in pain and function at a mean follow-up of 15.3 months [38]. Also in the hip joint, the addition of AMSCs to hip arthroscopy has been demonstrated to be a viable option in patients with symptomatic chondral defects [58]. Dall'Oca et al. first reported good outcomes using aAMSCs only in six patients with early HOA at short-term follow-up [41]. However, aAMSCs represent just one of the available alternatives for injections in early HOA. Other options are generally represented by HA and PRP, both with debatable results reported in the available literature. HA effects on joint and musculoskeletal degenerative diseases (i.e., tendinopathies) are based on both viscosupplementation and biological response [59, 60]. Although the intra-articular HA may have beneficial effects on pain at and beyond 12 weeks in knee OA, their use is still not recommended in HOA, according to the Osteoarthritis Research Society International (OARSI) guidelines [25]. In fact, highly conflicting findings were reported in most of the studies conducted on HA injections of the hip [61–64]. Although PRP use was recommended in case of failure of a previous conservative treatment in early knee OA by two recent consensus meetings conducted by the European Society of Sports Traumatology, Knee Surgery and Arthroscopy (ESSKA) ORthoBIologics InitiaTive (ORBIT) and the International Cartilage Repair Society (ICRS) [23, 24], its use is still controversial in HOA [65–68]. However, some reports underlined good outcomes in the first 3 months after its injection in selected young patients [66]. Interestingly, a different time-related effects of HA and PRP injections were demonstrated in a recent case-control study [69]. Indeed, the authors reported that patients treated with PRP injections presented an earlier pain relief and subsequent worsening. In contrast, those treated with HA presented a later efficacy but a constant improvement of pain during the follow-up [69]. Similar results were also reported by Battaglia et al. and Dallari et al. [70, 71]. Although some authors suggested that the injection of aAMSCs might be associated with good outcomes at middle-term follow-up [72], the time-related effects of aAMSCs were not investigated yet. Our study first analysed these effects on both function, pain and quality of life. Particularly, considering the categories emanated by Dworkin et al. on the measurement of pain relief in clinical trials, we obtained a clinical substantial pain relief (namely, > 50% of the baseline) in most of the patients [73]. Moreover, although to the best of our knowledge, no specific studies had been conducted on the minimum clinical improvement of OHS and SF-12 in injections for HOA, we reported a relevant result also in this field. Indeed, we reported an improvement of 15 points from the baseline of OHS. This result is far above the minimal clinically important difference threshold reported for THA (5.2 points) [74]. In our opinion, this latter must be remarkable, also considering the extremely negative effects of HOA on quality of life reported by Hampton et al. [75]. An analysis on the time-related effects of BM-MSCs in HOA was conducted by Mardones et al. in a study on multiple intra-articular injections [76]. The authors observed a constant increase in both pain relief and hip function during the entire follow-up period [76]. Although we did not perform a direct comparison between BM-MCs and aAMCs injections, we consider a data of interest that we observed a constant amelioration in hip function after a single injection of aAMSCs. Several mechanisms might explain the reason why a single injection of aAMSCs might lead to similar outcomes to those reported with multiple ones of BM-MSC. First, it has been demonstrated that adipose tissue contains a higher concentration of MSCs compared to the same volume of BM-MSCs [28]. Furthermore, aAMSCs have been reported to secrete more growth factors than BM-MSCs [77, 78]. Accordingly, aAMSCs present a better proliferative activity, migration, differentiation and senescence tolerance compared to BM-MSC [79, 80]. Finally, a higher immunosuppressive activity was reported with the use of aAMSCs [79]. In our opinion, these findings might suggest that aAMSCs could be a more valuable option in those circumstances where an immunomodulatory effect is more required, such as in HOA. Our population presented a high patients' satisfaction rate and an improved quality of life after the aAMSCs injection. Moreover, knowing the time-related effects of a single injection of aAMSCs in HOA could be extremely important to better understand its effectiveness and to improve patients' information need. Our study has some limitations, including the small sample size and the lack of a comparative group. Moreover, we do not have enough data to completely exclude some confounding variables (i.e., NSAIDs use after the procedure or the type of primary OA). However, the strict inclusion criteria (failure of the conservative treatment including NSAIDs' prolonged use) lead us to consider it meaningless for the present study. Although we cannot completely exclude that our results might be related to placebo effects, these are described to be time-dependent [81]. In particular, the placebo effect in HOA could be observed especially in the first 6 months after the start of a treatment [82]. Therefore, our longer follow-up make us confident that the results at 12 months could be mostly related to the proposed procedure.

Moreover, the present study reports the results in one of the largest cohort available in the current literature. Finally, the innovation of the procedure and the lack of a gold standard for the conservative treatment of HOA lead us to report these preliminary and promising time-related results.

## 5. Conclusions

The use of a single injection of aAMSCs in HOA significantly improved joint pain, function and patients' quality of life throughout the entire period of follow-up of 12 months. This procedure presented some advantages: easy to use, quick and minimally invasive technique and the absence of serious adverse effects. Despite the limited sample, the results of the present study showed that the proposed procedure was an effective treatment for HOA. However, further studies with larger samples and longer follow-ups are needed to confirm our data.

## Figures and Tables

**Table 1:** Indications for the injection of aAMSCS in HOA.

Diagnosis of HOA according to ACS criteria (pain in combination with either (1) hip internal rotation ≥ 15°, pain present on internal rotation of the hip, morning stiffness of the hip for ≤ 60 min and age > 50 years, or (2) hip internal rotation < 15° and an erythrocyte sedimentation rate (ESR) ≤ 45 mm/hour; if no ESR was obtained, hip flexion ≤ 115° was substituted)
Primary HOA
Failure of previous conservative treatment (NSAIDs and/or physiotherapy)
Age > 18 years
BMI > 18
Absence of: infectious joint disease, chondromatosis of the hip, malignancy
Absence of recent arthroscopic treatment or hyaluronic acid or other injections in the previous 6 months

**Table 2:** Baseline characteristics of the studied population.

Total no:	30
Age (year), mean (range)	58.7 (38–79)
Patient sex	
Male	12
Female	18
Kellgren–Lawrence stage	
Grade 1	3
Grade 2	18
Grade 3	3
Grade 4	6
Side	
Right	21
Left	9
Follow-up (months), mean (range)	11.4 (6–12)

**Table 3:** Mean VAS, OHS and SF-12 score.

	T0 (preoperative)	T1 (3-month F.U.)	T2 (6-month F.U.)	12-month F.U.
VAS	7.90	5.7	4.5^∗^	3.8^∗^
OHS	20.07	30.7^∗∗∗∗^	33.83^∗∗∗∗^	35.92^∗∗∗∗^°
MCS-12	42.28	46.75^∗∗^	48.01^∗∗^	48.15^∗^
PCS-12	28.97	36.86^∗∗∗^	40.90^∗∗∗∗^°	42.43^∗∗∗∗^°°

*Note:* PCS-12: 12-item Short Form Survey physical; MCS-12: 12-item Short Form Survey mental.

Abbreviations: F.U. = follow-up, OHS = Oxford Hip Score and VAS = Visual Analogue Scale.

^∗^ *p* < 0.05.

^∗∗^ *p* < 0.01.

^∗∗∗^ *p* < 0.001.

^∗∗∗∗^ *p* < 0.0001 vs. preoperative.

°*p* < 0.05.

°°*p* < 0.01 vs. 3-month F.U.

**Table 4:** Characteristics of the failed patients.

Patient I.D.	Sex	Age	OHS at T0	OHS at final F.U.	VAS at T0	VAS at final F.U.	PCS-12 at T0	PCS-12 at final F.U.	MCS-12	MCS-12 at final F.U.	Months between aAMSCs injection and failure
4	M	66	29	31	8	6	34.8	38.83	54.43	57.85	6
27	F	55	18	20	9	9	48.07	48.07	54,96	54.96	8
29	M	79	22	29	8	4	26.31	30.73	38.58	38.58	12

*Note:* PCS-12: 12-item Short Form Survey physical; MCS-12: 12-item Short Form Survey mental; aAMSCs: autologous-derived adipose mesenchymal stem cells.

Abbreviations: F.U. = follow-up, OHS = Oxford Hip Score and VAS = Visual Analogue Scale.

## Data Availability

The data that support the findings of this study are available on request from the corresponding author. The data are not publicly available due to privacy or ethical restrictions.
